# Trajectories of subject-interests development and influence factors in higher education

**DOI:** 10.1007/s12144-021-02691-7

**Published:** 2022-01-08

**Authors:** Steffen Wild

**Affiliations:** Chair of Psychological Diagnostics, Faculty 13 Rehabilitation Sciences, TU Dortmund University, Emil-Figge-Str. 50, 44227 Dortmund, Germany

**Keywords:** Interest development, Higher education, Trajectories, Subject-interests

## Abstract

It is a well-studied phenomenon, that throughout the course of studying at university, the motivation for the study program decreases. Correlation between motivation and learners’ behaviour, for example the learning process, achievement or, in the worst case, dropout exist. So there is a need for understanding the development of motivation in detail, like that of subject-interests, and for identifying influence factors, especially for higher education. This panel study examined the development of 4,345 students in higher education. Growth mixture models for subject-interests identify two classes of trajectories: “descending interest” and “continuously high interest”. In a next step, the analysis shows that gender, university entrance score, academic field and occupational aspiration influence membership of the classes. The results are discussed with respect to their consequences for education programs, but also with respect to possible new research questions.

## Highlights

• Two trajectory classes of subject-interest development in higher education are identified.

• Gender and academic major influence membership in the two different classes.

• The cognitive factors university entrance score and GPA affect the development of subject-interests.

• Occupational aspiration is an important factor for class affiliation.

## Introduction

A timeless challenge for education research is how to improve the academic performance of individuals (Hidi & Harackiewicz, 2000). Motivation is a key to understanding (Richardson et al., 2012) as it often decreases over time in education programs (Gaspard et al., 2020) and that this decrease in the worst case can lead to student drop out (Schnettler et al., 2020). To look at it in more detail, interest is seen as a crucial dimension within motivation theories that influences learning. Scientists have shown its impact on attention, goals and levels of learning (Hidi & Renninger, 2006; Renninger & Hidi, 2019). Further research results show that content-specific interests can be seen as an important factor in college students’ academic choices and performance (Harackiewicz et al., 2002).

The importance of interest is supported by other academic disciplines. For example, neuroscientists have detected interest as a motivator that influences learning and achievement and thus suggest that educators should focus on how they can best support their students’ interest development (Hidi, 2006). A reason is, that well developed individual interests can help individuals overcome a lack of ability and/or perceptual disabilities in math or reading (Renninger et al., 2002). Furthermore, teachers who recognize the potential benefits of increased academically relevant interests may be best positioned to enhance their students’ learning. Research data from educational psychology further supports this claim (Hidi, 2006).

Research on interest still results predominantly from cross-sectional studies (Dotterer et al., 2009). Yet, for an ontogenetic analysis of the development of interests it is important to consider intraindividual developmental processes (cf. Krapp, 2002). Occasionally, this is possible for researchers analysing longitudinal data. Another problem is that most studies on interest are situated in the field of primary and secondary education research. The preponderance of researchers reverts to the approaches and results of these studies. Longitudinal studies on higher education are the exception and can only be found very sporadically in this research field (Harackiewicz et al., 2002; Liebendörfer & Schukajlow, 2017).

This study extends previous research by examining the development of interest over time in a panel design. The major goal of this study is to analyze data based on the findings in primary and secondary school research and examine whether these effects also exist in higher education. Furthermore, I integrate new aspects in this study such as occupational aspiration, to provide a broader picture of the role of developing interest. Last but not least I try to find out whether there are not only two groups of either interested and uninterested people but also other intermediate forms with unspecific developmental trajectories.

## Interest

### Theoretical Assumptions and Delimitation

**Fig. 1:**
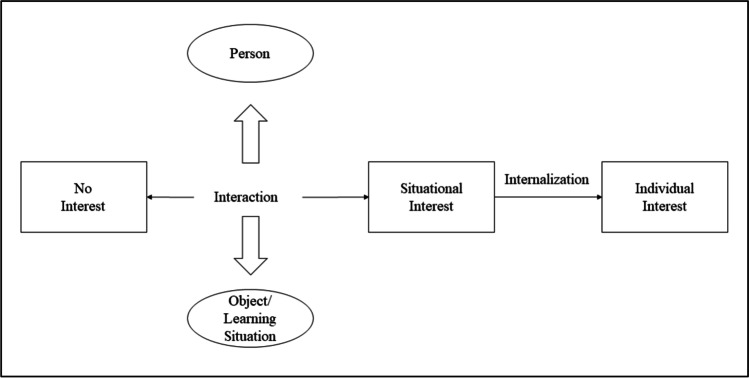
Main constituent parts of Person-Object-Theory of Interest (POI)

The importance of interest for education has been recognized since the late nineteenth century (Hidi, 2006). Today, one way to depict interest describes it as a special interaction with the environment, either a Person‐Object‐Interaction (leading to the development of “individual interest”) or a Person‐Stimulus‐Interaction (leading to “situational interest”) (Krapp, 2007). This approach is called Person‐Object‐Theory (POI) and is visualized in Fig. 1. Its assumptions are on the one hand that individual interest is a person’s characteristic and conceptualized as a stable personal disposition, and on the other hand that situational interest is based on interesting stimuli described as a momentary specific motivational/psychological state or object within a person. Both types of interest can be seen as a development and influence from each other (Pany et al., 2019). A deeper understanding of POI following Krapp (1992) and Schiefer et al. (2018) suggest that interest (e.g. subject-interest) has three components: First, there is the object of interest, which defines the concrete content of the interest (e.g. the content of the subject Economy). Second, there are actions of interest that are carried out to engage with the object of interest (e.g. reading a book or writing a text). And finally, there are concrete objects that are used to deal with the object of interest (e.g. a video or a poem).

According to Krapp (2002), this concept of interest also contains a combination of emotional and value-oriented components: the person assigns a personal value to the object of interest and feels positive emotions triggered by the sum of the object-related actions when dealing with the object. Especially with regard to the value component, it is also assumed that people with a stably developed interest identify with the object of interest and that it becomes part of their self-definition. While situational interest is only present in a specifically interesting situation, individual interest is anchored in the person's interest self-system.

To understand interest more precisely, researchers need to look at other motivational variables. The development of new interest starts with the triggering of attention to a specific content. An external impulse can help to raise the chance that an individual continues to deal with this content (Renninger & Hidi, 2019). Curiosity can also trigger a person’s attention, but while feeling curious, a person might encounter, and recognize, a knowledge gap (Loewenstein, 1994). Loewenstein’s theoretical framework of information gap holds that curiosity functions like other drive states, like hunger, which motivates eating. Building on this assumption, Loewenstein suggests that a small amount of information serves as a priming dose that increases curiosity. Consumption of information is rewarding, but, eventually, when enough information is getting, satiation occurs, and information serves to reduce more curiosity. There is an ongoing debate on the exact relation of interest and curiosity. According to Grossnickle (2016) a difference is that interest develops over time compared to curiosity. Adding to that, Renninger and Hidi (2016) work out that information-seeking processes as well as the basis and outcomes of the search differ. Curiosity is seen as a desire to seek and learn new information by exploring novel and uncertain environments. Individual interest focuses the motivation to seek and learn new information because it is linked to some form of existing knowledge, which then continues to develop. Further research shows a reciprocal relation between interest and goal setting (Harackiewicz et al., 2008), self-efficacy (Hidi & Ainley, 2008), and self-regulation (Sansone & Thoman, 2005). Renninger and Hidi (2019) conclude that these variables are distinct, and that in earlier phases of interest they may appear to be unrelated while in later phases of interest they are coordinated and mutually supportive.

This study conceptualises subject-interest as individual interest. According to Hoffmann (2002), subject-interest can be conceptualized in two different ways: on the one hand as interest in the topics of the respective subject and on the other hand as interest in the entire teaching of the subject—how it is taught and what is learned. Subject-interest in this research is conceptualized in the first manner. I define subject interest in this study as the match between personal interests and learning opportunities in one's own field of study (cf. Fellenberg & Hannover, 2006).

### Theoretical Framework for the Development of Interest

Findings in research about education programs often report the decrease in interest. For example, pupils enter elementary and secondary school with a high level of interest in individual school subjects. This interest declines throughout the course of their schooling (Dotterer et al., 2009; Frenzel et al., 2010, 2012). A variety of different assumptions are made to explain this phenomenon and react.

One possible explanation for these findings is that extracurricular areas of interest increase and compete with school interests (e.g. Hartinger & Fölling-Albers, 2002). Another suggestion, the stage-environment-fit approach (Eccles et al., 1993), emphasizes a mismatch between the needs and interests of young people and the supply structures of the school context. Finally, Daniels (2008) proposes that pupils undergo a process of differentiation and hence solely focus on a few areas of interest in their schooling time so that only selected subject areas of interest stay high.

Interest theories use different approaches to explain these findings. The framework of person object theory of interest distinguishes two interrelated subsystems: 1. emotional experiences and 2. conscious-cognitive factors (Krapp, 2005). These systems explain the start of an activity in a certain domain triggered by situational interest, and the continuous engagement in a specific object area because of stable individual interest. Emotional experiences based on a biological component. Here, emotions give a feedback on the organism's state of functioning in a situation. Conscious-cognitive factors refer to the process of rational–analytic intention. This is important when a person’s own controlled actions are responsible, in a consciously effortful way, in overcoming obstacles during a goal-oriented activity or an uninteresting, but significant task. When both subsystems are positive, interest develop.

**Fig. 2:**
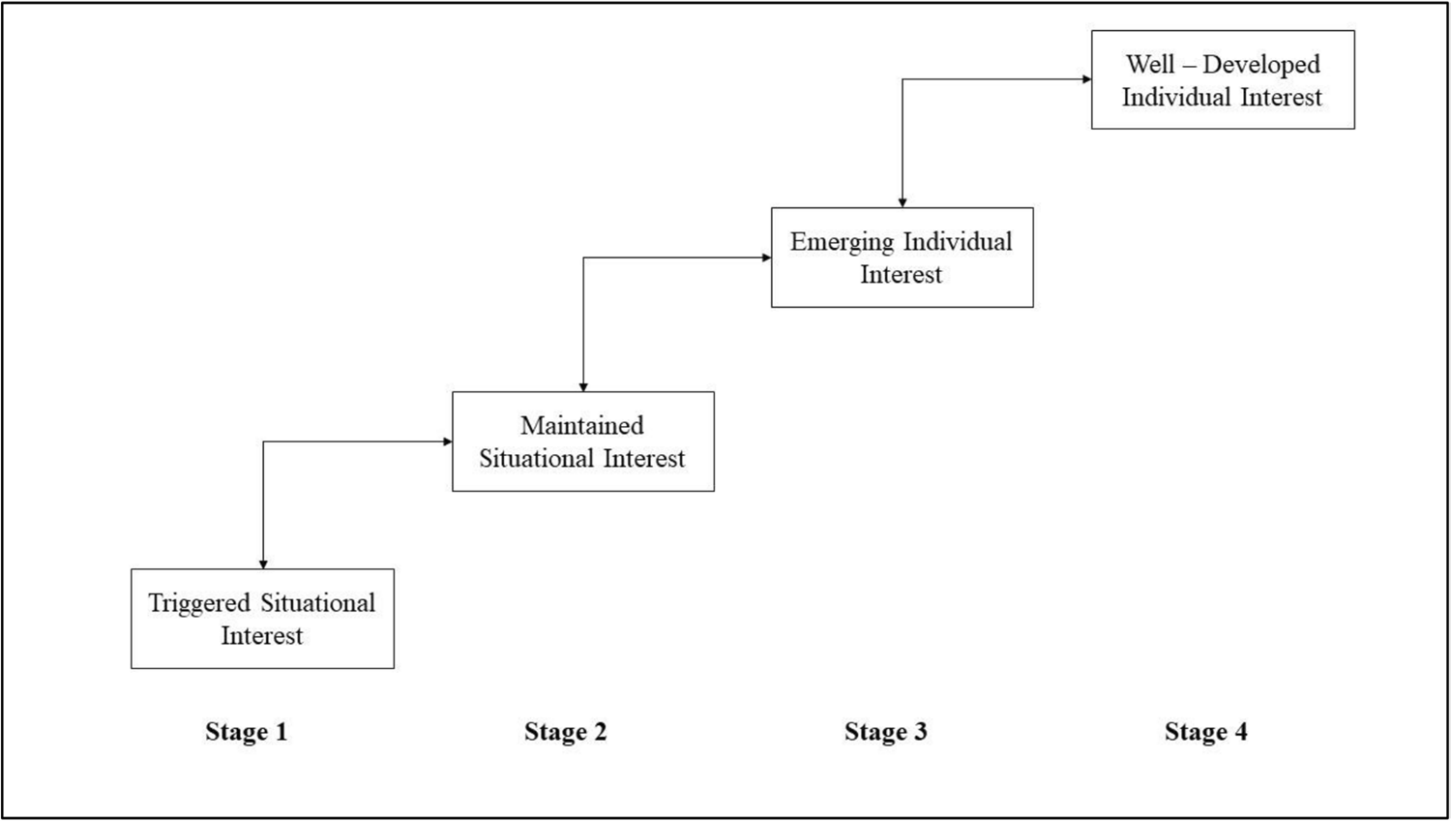
Levels of Interests in the Four‐Phase Model of Interest Development

According to the “Four‐phase model of interest development” (Hidi & Renninger, 2006; Renninger & Hidi, 2019), individual interest develops in four stages (Fig. 2). First, there is a triggered or “catch”‐component of situational interest: a person comes into contact with a stimulus that raises their attention. The second phase consists of maintaining situational interest or a “hold”‐component of situational interest: out of attention an experience develops that combines a growing sense of value with an epistemic orientation toward the content—the person is willing to know more about the object of interest. The third phase is seen as emerging individual interest. Here, the person develops positive feelings, has stored knowledge, and attaches a personal value to the interesting object. In addition to this, the person generates their own “curiosity” questions concerning the content of an emerging individual interest. The fourth and final phase is characterized as well‐developed individual interest. Here, a person engages with the object of interest against the background of their own set of values, increases the stored knowledge and starts to search and create answers to their own curiosity questions.

In summary, the development of interest starts with the triggering of attention and is followed by searching for information. In earlier phases, interest development can be facilitated by the structure of the environment or by interacting with other people. In contrast, in later phases, interest is built on content knowledge. However, in any phase of interest development, the level of interest can be expected to plateau or fall. This happens if individuals have no opportunity to engage with content that allows them to further develop their understanding. Moreover, problems for the development of interests arise when individuals maintain competing interest or if they do not receive the support they need to make meaningful connections to content (Renninger & Hidi, 2019).

### Current State of Research on Interest in Education

Research on interest exists almost solely for primary and secondary education (Renninger & Hidi, 2019; Schiefele, 2009). For higher education, on the other hand, publications are rare. Fortunately, longitudinal studies have been used more often in the scientific discourse as numerous panel studies have been initiated in recent years (Blossfeld et al., 2019). The issue of diminishing interest over time, which has already been discussed in detail in the previous sub-chapter, is seen as important in science. Research on higher education confirm this (Xu et al., 2021). Here, other topics, such as performance or academic choices, are also often considered when dealing with interest (Schiefele, 2009).

Krapp (2002) and Schiefele (2009) report that subject-interest development is affected by subject areas, context conditions, school type and gender. This research is very focused and hard to generalize. Todt et al. (1974) show a decreasing interest for girls throughout secondary school for zoological and botanical topics in biology, but an increased interest in topics related to human beings and ecology biology. Other research in subject areas shows a low interest in physics teaching within a scientific context (with an emphasis on the validity of general physical laws) and a strong interest when the teacher is able to relate physical principles and facts to practical problems and the students’ everyday life (Hoffmann & Lehrke, 1986; Hoffmann et al., 1985). According to Høgheim and Reber (2019) girls report lower levels of individual interest in mathematics than boys do. A slight trend reported by Krapp (2002) implies that girls’ interests decrease faster than boys’.

The situation is different for the link between interest and performance. Schiefele et al. (1993) report a correlation of *r* = 0.30 in a meta-analysis. However, simple correlations are used for this analysis. Schiefele (2009) argue that in the current empirical state of research on interest the causal direction between interest and achievement remains an unresolved issue. Longitudinal research by Maurice et al. (2014) shows effects of achievement on interest for the 3rd Grade. Reciprocal instead of unidirectional effects between interest and achievement are reported by Scherrer et al. (2020). Schiefele (2009) concludes for lower secondary school level that interest is either a nonsignificant or weak antecedent of achievement, while for higher education, Rotgans and Schmidt (2011) show effects of interest on achievement.

Academic choices are also related to interest. Whereas in lower secondary schools, students’ motivation is mostly regulated by extrinsic incentives and values, in upper secondary school, interest gains more influence on the regulation of learning activities and highly interested students more often choose an advanced course. These findings are in line with research on academic choices by Bong (2001), Durik et al. (2006) and Eccles (1983), who presented evidence that the effects of motivational characteristics on academic choices are more substantial than those of achievement or learning. Moreover, the theoretical framework of the Wisconsin model (Hauser et al., 1983; Sewell et al., 1969) from social psychology assumes the importance of social origin, occupational aspirations and academic performance for educational attainment and outcome. Furthermore, empirical research underlines the existence of such effects (Kim et al., 2019; Sabates et al., 2011; Yates et al., 2011).

Researchers in higher education have often called for analyzing students over an extended period of time (Xu et al., 2021). But research in this field has mostly focused on evaluating learning gains and much less frequently on estimating growth rates of individual constructs (Coertjens et al., 2017; Kyndt et al., 2015) and developmental relationships between constructs (Kyndt et al., 2019). This situation highlights a significant challenge for research on students’ motivational development, especially for the construct interest, in higher education. In Europe exist no consistent findings according to this specific research field. Liebendörfer and Schukajlow (2017) published results, with a small sample size (N = 92) from lower secondary school teachers, showing that students’ interest in mathematics remained stable during the first academic year in Germany. Xu et al. (2021) show for students in Educational Sciences, Speech Pathology and Audiology in Belgium a decreasing of statistic interest over time by using a latent growth curve analysis for analysing data over two years. Furthermore, this study reports, that the rate of decrease in interest was positively associated with rates of growth in cognitive competence and utility value. However, the different development of subgroups within the sample was not analyzed. Further longitudinal studies on interest in the higher education sector indicate that mastery goals, e.g. the desire to develop new skills (Ames & Archer, 1988), are unrelated to academic performance but that they predict interest in the course. Students who adopted work avoidance goals report less subject-interests in psychology (Harackiewicz et al., 2002). Analyses for different developing groups of trajectories in subject-interests are not consistent. Research based on higher education is not well develop and as a consequence I use studies focused on elementary and secondary school. Research of Musu-Gillette et al. (2015) analyzes trajectories of interest in math from 4th grade to the 2nd year of college. Results show three latent classes with a curvilinear trajectory. The first class is called “High Interest Trajectory” (40 percent of the sample) starts with the highest reported levels of interest in math. That interest declines over time, especially between 6th grade and 10th grade, but shows an increase from late high school on into college. The trajectory could be interpreted as a u-shaped change. The second latent class is called “Slow Decline Interest Trajectory” (22 percent of the sample) and students in this class begin with moderate levels of interest in math in elementary school. Persons in this class show a decline in their reported interest over time with a curvilinear trend and stagnation in high school and into college. For the third latent class, “High Self-Concept Trajectory” (38 percent of the sample), initially high levels of interest in math, which decline rather steeply over time, are reported. In contrast, Schiefer et al. (2018) identify five different latent classes for subject-interests in German (native language), mathematics and English at grade 4 to grade 11. Taken together, this underlines the importance of studying students’ interest development.

### The Present Study

Drawing on the Wisconsin model, the POI and a four‐phase model of interest development, this study examines the trajectories in subject-interests in higher education. It aims at establishing different latent classes and linking these classes of subject interests to academic performance, demographic conditions, learning conditions and career choices. This study uses the well-known growth mixture modeling (GMM) to investigate qualitatively distinct trajectory classes (Guo et al., 2018; Musu-Gillette et al., 2015; Wang et al., 2017). GMM analyzes not only an average trend in the data, but also interindividual differences between student groups as well as intraindividual differences. Therefore, GMM is considered an appropriate approach for this study, as it allows to account for potential heterogeneity in developmental trajectories between groups of students as well as intraindividual differentiation across domains.

Two research questions are investigated. The first question is: “Can qualitatively different latent trajectory classes of subject-interest be identified?”. Because of the scarcity of previous research on this topic no specific predictions about the number and shape of such trajectories are made. However, I expect to find classes in which subject-interest remains high or decreases by controlling for the cognitive factors (Protsch & Solga, 2015), measured by Grade Point Average, and the context factor Quality of instruction (cf. Gaspard et al., 2020). I expect different trajectories against the background of the four‐phase model and the POI, because in earlier phases of a study program interest develops through the formal structure of the environment and through interacting with other people. In the last years of a study program interest is built on relatively open structures, opportunities and support, which students use in different ways.

The second question is: “Are individual (gender, occupational aspiration), cognitive (university entrance score), family background (social origin) or academic field factors related to affiliating with a specific latent class?” Based on previous research on links between these variables and the development of students’ subject-interest, I expect that high university entrance score and occupational aspiration predict higher levels of subject-interest as predicted in the Wisconsin model. For academic field, social origin and gender, no directional hypothesis is assumed, since, so far, no consistent research results on their links to subject-interest development have been attained.

This study closes a research gap by analyzing the development of subject interest in the whole bachelor program of students in higher education. Similar research that focuses this topic over such long periods of time with a similar number of measurement points as well as a similarly large sample size and for these academic majors is not known.

## Methods

### Participants and Procedure

The present research uses data from the panel study “Study Process – Crossroads, Determinants of Success and Barriers during a Study at the DHBW” from 2016 to 2019 (Deuer et al., 2020). The study is specifically designed to explore determinants of academic success in cooperative education at Baden-Wuerttemberg Cooperative State University (DHBW). These bachelor’s degree programs are made up of 210 ECTS (European Credit Transfer System) credits in six semesters (three years) and every three months, a cooperative student rotates between academic training at the university and workplace training at the company (Wild & Alvarez, 2020). Participation have been voluntary and privacy policy is protected. Every fiftieth student who answered more than one question received a 10 € coupon as an incentive for participation.

The numbers of students have been continuously rising since 2007 and as a consequence this study is done during the years 2016 and 2019 (Destatis, 2020, p. 31), including for cooperative education (AusbildungPlus, 2020, p. 11), and student dropout has become increasingly relevant as a topic in higher education research and policy (Neugebauer et al., 2019; Wild & Schulze Heuling, 2020). Four cohorts are included in this four-year study, since there is only a chance to get enrolled one time per year. Data collection is conducted once a year for economic reasons, resulting in four waves of data collection. Differences in the data sets are analyzed by measurement invariance and the results are presented in the following chapter “Measures”.

For the present analyses, data from all students who reported their subject-interest more than one time in the four waves is used. Every year, all 34,000 enrolled students at DHBW are invited to participate in the survey by two emails, separated by a two-week interval, which included a link to a questionnaire. Panel wave 1 is conducted in summer 2016 (response rate 19.7 percent), panel wave 2 in spring 2017 (response rate 18 percent), panel wave 3 in spring 2018 (response rate 24.3 percent) and panel wave 4 in spring 2019 (response rate 22 percent). A total of 4,345 students (58 percent female) from four cohorts (n = 565 for Cohort starting the study program in October 2014; n = 1,432 for Cohort starting the study program in October 2015; n = 1,526 for Cohort starting the study program in October 2016; n = 822 for Cohort starting the study program in October 2017) participated and contribute to the estimation of the GMM. Table 1 gives an overview of the collected data for each wave and cohort. In cohort 2015 and cohort 2016, more than 1,000 participants per wave take part in the study. In cohort 2014 only 565 students and in cohort 2017 822 participated. Range of the means in age is in cohort 2016 with *M*_*wave2*_ = 21.71 (wave 2) and *M*_*wave4*_ = 23.94 widest. Standard deviation ranges from *SD* = 2.71 (cohort 2015 in wave 2) to *SD* = 3.18 (cohort 2016 in wave 4). Attrition is a huge problem. Calculations show that only 512 students participated in all waves from wave 1 to wave 3 during their regular enrolment time of three years.

**Table 1:** Sample description by cohort, and age at each panel wave

Time and Variable	Cohort 2014	Cohort 2015	Cohort 2016	Cohort 2017
Panel Wave 1 (July 2016)				
*n*	565	1,049		
*M*	23.12	22.04		
*SD*	3.26	3.02		
Panel Wave 2 (March 2017)				
*n*	565	1,090	1,074	
*M*	23.79	22.67	21.71	
*SD*	3.26	2.71	2.93	
Panel Wave 3 (March 2018)				
*n*		1,144	1,312	822
*M*		23.71	22.78	21.78
*SD*		2.99	2.91	3.16
Panel Wave 4 (March 2019)				
*n*			1,138	822
*M*			23.94	22.78
*SD*			3.18	3.16

The average age was computed as of January 1 of each year shown (e.g., January 1, 1988)

### Measures

#### Subject-Interest

The subject-interest is measured with a modified instrument by Fellenberg and Hannover (2006) in every wave. Reliability on items of a 5-point Likert scale with values ranging from 1 (strongly disagree) to 5 (strongly agree) is excellent for all four waves (ω = 0.89–0.91). The modification of the instrument of originally 13 items is necessary for reasons of, because of test efficiency and research group discussion of better face validity. Table 2 shows the original instrument and the items used in this study. As a next step, I investigate the cut-off criteria by Chen (2007) by χ^2^-Test and Δ CFI ≤  − 0.005 in combination with Δ RMSEA ≥ 0.010 for the level of measurement invariance (Table 3). Using χ^2^-Test is problematic, because χ^2^ increases in power to reject the null hypothesis as the sample size increases. Having a larger total sample, this may lead to over-rejection of measurement invariance tests if the change in χ^2^ is the only criterion used to evaluate fit (Putnick & Bornstein, 2016). The χ^2^-Test in every model comparison is significant by *p* < 0.001. According to the benchmarks of Δ CFI ≤  − 0.005 and Δ RMSEA ≥ 0.010 for the level of measurement invariance, scalar invariance for the factors academic year and panel wave is indicated.

**Table 2:** Formulations of the Items in original Instrument (Fellenberg & Hannover, 2006) and used in this research for the scale subject interest

Item formulation
My field of study matches with my interests. *
I cannot imagine a more interesting subject than my field of study. *
My subject is exactly the right one for me. *
For me, dealing with the content of my subject is more of a frustration than a pleasure. *(-)
I enjoy dealing with topics in my subject
I often think about certain topics in my field of study
I enjoy exchanging ideas with others about topics in my subject. *
I have doubts about whether my subject really matches my interests. *(-)
I enjoy dealing with certain questions and problems in my field of study.*
The subject I study does not necessarily reflect my main interests. (-)
My subject of study is also my hobby. *
The interest in my subject of study is not excessively strong in me. *(-)
Actually, I am more interested in other subject contents than in those of my subject. (-)

Presented are translations of the original German items that are not yet validated in the English language; * = used in this research; (-) = inverse item

**Table 3:** Measurement invariance for the scale subject interest on four panel wave and academic year (n = 8,547)

	χ^2^	*df*	χ^2^/*df*	Δ χ^2^	Δ *df*	*p*	CFI	RMSEA	Δ CFI	Δ RMSEA
Academic year										
Configural Invariance	1102.3	69	15.98			< .001	.969	.078		
Metric invariance	1154.1	85	13.58	51.817	16	< .001	.968	.072	-.001	.007
Scalar invariance	1274.2	101	12.62	120.124	16	< .001	.965	.069	-.003	.003
Panel wave										
Configural Invariance	1100.1	92	11.95				.970	.077		
Metric invariance	1152.1	116	9.93	51.949	24	< .001	.970	.070	-.001	.008
Scalar invariance	1308.0	140	9.34	155.905	24	< .001	.966	.068	-.004	.002

Satorra-Bentler-scaled χ^2^-difference test. Academic year: one (n = 3,408), two (n = 2,797), and three (n = 2,342). Panel wave: one (n = 916), two (n = 1,293), three (n = 3,715), and four (n = 2,623)

#### Time

The information regarding the time of measurement is obtained from the survey software. To be able to model the time period in the study program to the exact day in the statistical model, the survey date was compared with the participants’ date of starting the study program.

#### Occupational Aspiration

To measure occupational aspiration of the students, this study employs a proxy variable. The item text is “The subject I am studying has been my "desired subject" from the very beginning.”. A 5-point Likert scale with values ranging from 1 (strongly disagree) to 5 (strongly agree) is used and the data was collected one time for each cohort.

#### Gender

The university’s administration provides the data for gender with male and female. I do not receive any data for gender diverse persons. The study matches this data to the collected data from the survey.

#### Academic Major

The test persons are enrolled in the three academic majors of Economy, Engineering and Social Work. These data are obtained from the university administration and matched to the survey data. Economy and Engineering is chosen, because those are the academic majors in Germany with the highest number of students enrolled in 2020 (Destatis, 2020, p. 31). Social Work is integrated in this research, because a shortage of skilled workers is expected in this field (Vogler-Ludwig et al., 2016). These academic majors vary in their didactics and teaching–learning methodologies. For example, Economy uses management simulations. Engineering uses technical laboratories, for example in the context of materials science. Social Work strongly reflects its works in the context of case management. Differences in the academic majors, like Economy and Engineering, exists for example in the basic needs from Self-Determination Theory (Wild & Neef, 2019).

#### Social Origin

Social origin is measured via parental education. This study distinguishes three origin groups: “low” if mother and father complete a lower or no school leaving certificate, “medium” if at least one parent gained a higher education entrance qualification and “high” if at least one parent has a degree in higher education. This data is collected one time during the panel survey.

#### University Entrance Score and GPA

German university entrance scores in the survey vary between 1 (equivalent to A in Great Britain and United States of America) and 4 (equivalent to E (GB) or D (US)) and GPA varies between 1 (equivalent to A in Great Britain and United States of America) and 5 (equivalent to E (GB) or D (US)). The date is recoded for better interpretation so that 5 is the best score and 2 (university entrance scores) or 1 (GPA) are the lowest score. Data for the GPA and the university entrance scores is provided by the university administration for GPA.

#### Quality of Instruction

An adjusted scale by Thiel et al. (2008) measures Quality of instruction with eight items that vary between 1 (strongly disagree) and 5 (strongly agree). The reliability in all four waves shows good values (ω = 0.81–0.82; item example: In general, the courses are well structured.). The data is collected in every wave.

### Data Analysis Strategy

To achieve the main objective of the present study, I use GMM for analysing research question 1 (Fitzmaurice et al., 2009). A hierarchical logistic regression is estimated to test the assumptions of research question 2 (Tabachnick & Fidell, 2013). Each step is described in more detail in the following section. According to Richard et al., (2003; p. 339) I interpret the effect size of *r* = 0.10 – 0.19 as small, *r* = 0.20 – 0.29 as medium and *r* ≥ 0.30 as large in the first paragraph of the chapter results.

All analyses are conducted with R version 3.6.2. The reliability analysis of ω (McDonald, 1999) is done with the package “MBESS”. The GMM is analyzed with the package “lcmm” and logistic regression analyses are conducted with the package “margins”.

To address the first research question, this study follows the proposed approach of Ram and Grimm (2009) for the analysis of the GMM. Thus, this study examines the shape of growth over time using growth curve analyses for one single group. Both linear and quadratic growth models are tested. As a next step, a model specification based on previous models is conducted to identify unobserved subgroups of individual trajectories. The analysis compares models with increasing numbers of classes. Comparisons across models are conducted with the Akaike information criterion (*AIC*), the Bayesian information criterion (*BIC*) fit statistics, and the sample-adjusted BIC (*SABIC*), with smaller values indicating superior fit to the data. The entropy value is measured (ranging from 0 to 1) as an indicator of classification accuracy. Values > 0.70 indicate a good classification accuracy (Reinecke, 2006) and the diagonal of the average latent class probabilities for most likely class membership near 1 (Jung & Wickrama, 2008). Finally, plots of the group trajectories are inspected for the plausibility of the results.

Subsequently, this study investigates differences in the sets of student characteristics and outcomes across the previously identified classes (Research Questions 2). For this, hierarchical logistic regression and Likelihood Ratio Tests are used (Glover & Dixon, 2004). Average Marginal Effects (*AME*) are estimated, because this procedure leads to satisfactory results in many different scenarios (Best & Wolf, 2012; Mood, 2010).

The results for estimated fixed effects model are reported by four decimal places (ten thousandths). The reason for this is, that the effect is small yet still positive or negative and does not actually include zero. Only two decimal places with zero and statistically significant results would confuse the reporting. Especially in the case large sample size, where almost everything will be significant.

The percentage of missing values of each variable in the dataset of the GMM is below 0.1 percent. A Missing Values Analysis indicates that Little’s (1988) test of Missing Completely at Random (MCAR) is not significant (*χ*^*2*^ = 2.071, *df* = 2, *p* = 0.36). For the logistic regression dataset, the percentage of missing values on each variable vary between 0 and 2.9 percent. Little’s (1988) test is not significant here, either (*χ*^*2*^ = 16.826, *df* = 25, *p* = 0.88). Thus, there is no evidence that the data was not MCAR. The missing data is estimated using the R Package “Amelia” and the EMB algorithm method, which combines the classic Expectation Maximization (EM) algorithm with a bootstrap approach (Honaker et al., 2011), using m = 5 imputed datasets. GMM models are estimated based on z-scores.

## Results

Table 4 provides descriptive statistics and correlations (*r*) for all variables across academic years. The means on the 5-point Likert scale (1 = “strongly disagree” to 5 “strongly agree”) range between *M* = 3.42 for quality of instruction and *M* = 3.92 for GPA – each in the third academic year. As a next step, I inspect the correlations. According to Richard et al. (2003), large correlation effect sizes exist between occupational aspiration and subject-interest (*r* = 0.31 to *r* = 0.42). In this study, the measurement for the third academic year also shows medium and large effect sizes of the correlations for subject-interest and quality of instruction between *r* = 0.27 and *r* = 0.34. The two performance measurements of university entrance score and GPA correlates with *r* = 0.33 and *r* = 0.42.

**Table 4:** Descriptive statistics and correlations (*r*) among metric key variables between academic years (n = 1,450 ‒ 4345)

	1	2	3	4	5	6	7	8	9	10	11
1. Subject-interests (Academic year 1)											
2. Subject-interests (Academic year 2)	‒.72***										
3. Subject-interests (Academic year 3)	‒.65***	‒.76***									
4. Quality of instruction (Academic year 1)	‒.34***	‒.26***	‒.23***								
5. Quality of instruction (Academic year 2)	‒.21***	‒.31***	‒.24***	‒.56***							
6. Quality of instruction (Academic year 3)	‒.17***	‒.24***	‒.27***	‒.48***	‒.64***						
7. GPA (Academic year 1)	‒.10***	‒.12***	‒.13***	‒.06**	‒.05**	‒.02					
8. GPA (Academic year 2)	‒.12***	‒.15***	‒.16***	‒.05*	‒.08***	‒.05*	‒.86***				
9. GPA (Academic year 3)	‒.09**	‒.16***	‒.17***	‒.04	‒.07**	‒.03	‒.82***	‒.94***			
10. Occupational aspiration	‒.42***	‒.38***	‒.31***	‒.11***	‒.08***	‒.08***	‒.00	‒.02	‒.05**		
11. University entrance score	‒.04*	‒.05	‒.02	‒.06**	‒.05**	‒.08***	‒.33	‒.39***	‒.42***	‒.01	
*M*	3.70	3.59	3.56	3.70	3.50	3.42	3.85	3.86	3.92	3.90	3.84
*SD*	3.66	3.74	3.77	3.53	3.58	3.60	3.60	3.52	3.45	1.16	3.59

*GPA* Grade Point Average; †*p* < .10; *p < .05; **p < .01; ***p < .001

Step 1 of the analysis involves exploring the functional form of the growth curve in students’ subject-interests across the full sample. Table 5 shows the results of the two estimated fixed-effects models without (Model 1) and with control variables (Model 2). I see that the linear slope factor for time is negative for Model 1 (*β* = ‒0.0218) and Model 2 (*β* = ‒0.0095). Both models are significant *p* < 0.05. The quadratic slope factor for time is seen as very small. In Model 1 it is *β* = 0.0004 and significant (*p* < 0.001). In Model 2 the effect is *β* = 0.0002 and marginally significant (*p* < 0.1). The effects for the control variables GPA (*β* = 0.1672; *p* < 0.001) and quality of instruction (*β* = 0.3739; *p* < 0.001) are larger, than the time effect. Because of these results a quadratic growth factor for subject-interest is assumed and the two control variables in the model are kept for further analysis.

**Table 5:** Fixed-effects models for changes in subject-interests in study program (full sample)

	Model 1	Model 2
	*β*	*β*
Time	‒.0218 (.0049)***	‒.0095 (.0046)*
Time (quadratic)	.0004 (.0001)***	.0002 (.0001)†
GPA		.1672 (.0134)***
Quality of instruction		.3739 (.0123)***
Intercept	3.8477 (.0319)***	1.7456 (.0747)**
Number of Persons	4,345	4,345
Number of Observations	9,581	9,581

Standard errors are shown in parentheses; *β* = standardized beta coefficients; *GPA* Grade Point Average; †*p* < .10; *p < .05; **p < .01; ***p < .001

Step 2 of the analyses addresses research question 1 concerning how many classes of students’ trajectories can be identified. The study estimates growth mixture models varying from a one-class solution to a five-class solution. Table 6 summarizes the results. *AIC*, *BIC* and *saBIC* decrease in all estimated models, which indicates a solution with only a few classes. Entropy varies between two classes and four classes in a range from 0.72 to 0.74. The researcher finally chose a two class solution, because its entropy of 0.74 is the highest of all class solutions, an appropriately large class size ranging from 24 to 73 percent—the smallest class sizes in the other estimated models could be seen as marginal groups or outliers—and highest large average latent class probabilities for the most likely latent class membership between 89 and 94 percent.

**Table 6:** Fit indices from estimated growth mixture models

Model	*AIC*	*BIC*	*SABIC*	Entropy	Minimum size of class in percent	maximum size of class in percent	minimum average latent class probabilities for most likely latent class membership	maximum average latent class probabilities for most likely latent class membership
1-Class	26043.95	26082.21	26063.14	-	-	-	-	-
2-Class	23612.01	23688.53	23650.40	.74	27	73	89	94
3-Class	22550.95	22665.73	22608.54	.72	12	53	86	90
4-Class	22113.53	22266.57	22190.31	.73	5	52	83	88
5-Class	21998.20	22189.50	22094.17	.69	2	43	75	87

*AIC* Akaike information criterion, *BIC* Bayesian information criterion (*BIC*), *SABIC* Sample-adjusted Bayesian information criterion

**Fig. 3:**
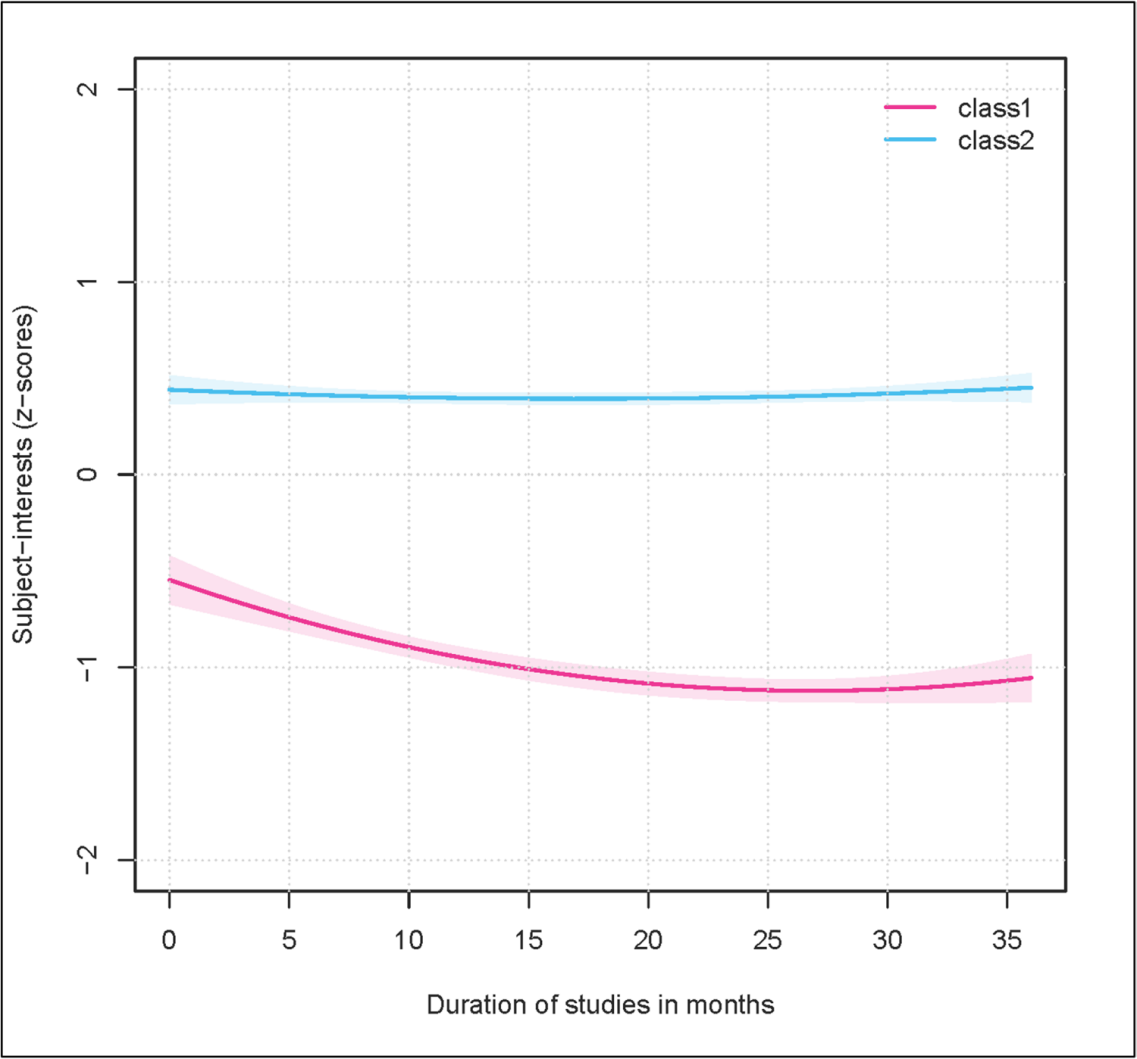
Estimated average trajectories of the growth mixture modeling (2-Class solution). *Notes:* Sample size of class 1 “descending interest” (below trajectory) is 27 percent; Sample size of class 2 “continuously high interest” (above trajectory) is 73 percent

Figure 3 plots the result for the two class solution. I use z-scores in our plot. The trajectories for class 1 (27 percent of the sample) starts at nearly *z* = ‒0.50 and decreases in the following months. In month 15, the score is under *z* = ‒1. Although the score rises again from around the 20th month, it never rises above *z* = ‒1 in the subsequent period. It is speculated that subject-interests will increase, because of the thesis here. The trajectory for class 2 (73 percent of the sample size) starts at about *z* = 0.50 with less changes. This trajectory is always above the mean with a z-score > 0. As a consequence of these results, I name class 1 “descending interest” and class 2 “continuously high interest” for further analyses.

Table 7 shows the estimated development trajectories of the two classes solution as fixed-effects models. The intercept for the class “descending interest” is *β*_*0*_ = ‒0.5451 and for the class “continuously high interest” *β*_*0*_ = 0.4401. The development of the class “descending interest” shows a decreasing slope (*β* = ‒0.0427; *p* < 0.001) with a quadratic trend (*β* = 0.0008; *p* < 0.001). In contrast, the class “continuously high interest” shows a smaller negative effect (*β* = ‒0.0055; *p* > 0.10). GPA (Class “descending interest”: *β* = 0.1239; *p* < 0.001; Class “continuously high interest”: *β* = 0.0867; *p* < 0.001) with larger effect against time and quality of instruction (Class “descending interest”: *β* = 0.3489; *p* < 0.001; Class “continuously high interest”: *β* = 0.2158; *p* < 0.001) with largest effect in the whole model are significantly positive for these two variables and both classes. This result underlines the importance of these variables from a theoretical point of view, for their practical implications and finally for the estimated model.

**Table 7:** Fixed-effects models for changes in subject-interests in study program (2-Class solution)

	descending interest	continuously high interest
	*β*	*β*
Time	‒.0427 (.0079)***	‒.0055 (.0047)
Time (quadratic)	.0008 (.0002)***	.0002 (.0001)
GPA	.1239 (.0174)***	.0867 (.0099)***
Quality of instruction	.3489 (.0177)***	.2158 (.0096)***
Intercept	‒.5451 (.0643)***	.4401 (.0379)***
Number of Persons	4,345	
Number of Observations	9,581	

Standard errors are shown in parentheses; table shows standardized beta coefficients; *GPA* Grade Point Average; †*p* < .10; *p < .05; **p < .01; ***p < .001

**Fig. 4:**
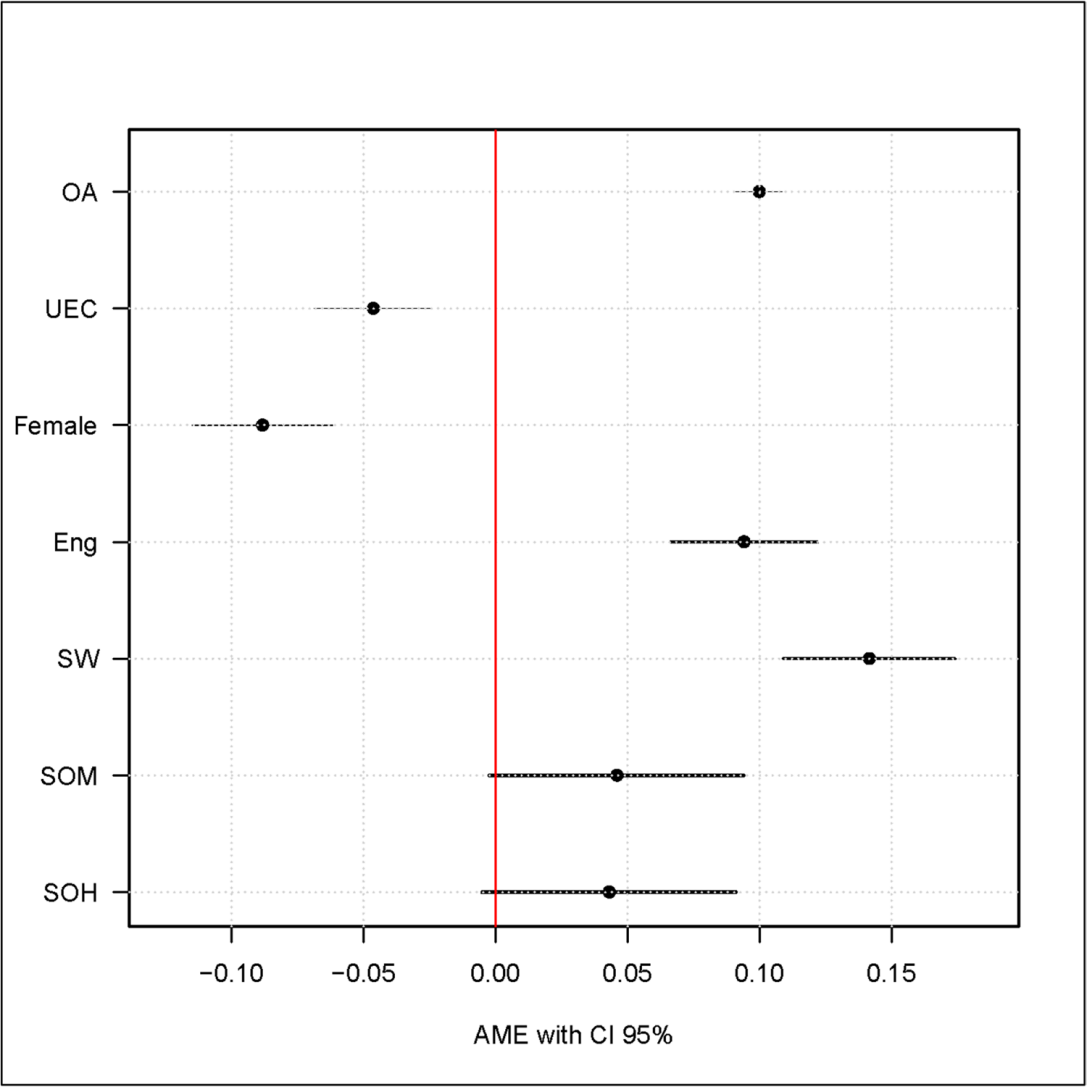
Coefficient plot for prediction of membership in class “continuously high interest” based on logistic regression (n = 4,345). *Notes:* table shows *AME* Average Marginal Effect, *OA* Occupational aspiration, *UEC* University entrance score, *Eng* engineering (ref. economy), *SW* social work (ref. economy), *SOM* Social origin middle (ref. Social origin low), *SOH* Social origin high (ref. Social origin low)

To work on research question 2 and to identify individual, cognitive and background factors for membership in different classes, this study uses logistic regression analysis, which is depicted in Table 8. To check the robustness of the results, I systematically extend the estimated models by adding variables. Results of Model 1 (*McFadden's adj. R*^*2*^ = 0.13; *Nagelkerke R*^*2*^ = 0.19; *Cox & Snell R*^*2*^ = 0.12) show that occupational aspiration (*AME* = 0.10; *p* < 0.001) and the academic majors of social work (*AME* = 0.16; *p* < 0.001) and engineering (*AME* = 0.08; *p* < 0.001) influence belonging to the class “continuously high interest”. However, there is a significant effect for female against male participants being in the class “descending interest” (*AME* = ‒0.09; *p* < 0.001). These effects remain almost unchanged in the other estimated models. Cognitive factors depict by the university entrance score are integrated in Model 2. However, this effect is negative (*AME* = ‒0.05; *p* < 0.001) and students with better scores belong to the class “descending interest”. A likelihood ratio test in between Model 1 and Model 2 shows a modest improvement in model fit (*McFadden's adj. R*^*2*^ = 0.12; *Nagelkerke R*^*2*^ = 0.20; *Cox & Snell R*^*2*^ = 0.13; *χ*^*2*^ (1) = 15.99, *p* < 0.001). Model 3 includes family background factors. The effect of social origin “middle” (*AME* = 0.05; *p* < 0.10) and social origin “high” (*AME* = 0.04; *p* < 0.10) is not as high as the other variables in the models and only marginally significant. A likelihood ratio test between Model 2 and Model 3 shows no improvement in model fit (*McFadden's adj. R*^*2*^= 0.12; *Nagelkerke R*^*2*^= 0.20; *Cox & Snell R*^*2*^ = 0.14; *χ*^*2*^ (2) = 3.47, *p* > 0.10). Figure 4 plots the results of Model 3 in Table 7 using a coefficient plot.

**Table 8:** Logistic regression for prediction on membership on class “continuously high interest” (n = 4,345)

	Model 1	Model 2	Model 3
	*AME*	*AME*	*AME*
Occupational aspiration	.10 (.01)***	.10 (.01)***	.10 (.01)***
Gender: female (ref. male)	‒.09(.01)***	‒.09(.01)***	‒.09(.01)***
Academic major (ref. economy)			
social work	.16(.02)***	.14(.02)***	.14(.02)***
engineering	.08(.01)***	.09(.01)***	.09(.01)***
University entrance score		‒.05(.01)***	‒.05(.01)***
Social origin (ref. low)			
middle			.05 (.03)†
high			.04(.02)†
*Cox-Snell R*^*2*^	.13	.13	.14
*Nagelkerke R*^*2*^	.19	.20	.20
*McFadden's adj. R*^*2*^	.12	.12	.12

Standard errors are shown in parentheses; table shows *AME* Average Marginal Effect. †*p* < .10; *p < .05; **p < .01; ***p < .001

## Discussion

This study is one of the first studies to examine developmental processes of subject-interest in higher education in a longitudinal perspective analyzing inter- and intraindividual differences. The first major aim of this study is to identify different types of trajectories. In a subsequent step, determinants are tested that influence the membership in these trajectory types as second major aim.

This research identifies a decreasing subject-interest over all observations. GMM models are estimated for analysing research question 1 detect two classes of trajectories. Class 1, classify as “descending interest”, shows a decreasing curve from the beginning of university on, and a slow rise from the 20th month onward. This curve is in line with previous findings from motivation research (Dotterer et al., 2009; Frenzel et al., 2010). The second class, characterize as “continuously high interest”, shows an almost parallel curve above the overall mean. The interest in this class stays on a continuously high level. Gaspard et al. (2020) publish similar results for elementary and secondary school, with two trajectory lines for motivational variables. Consequently, the results replicate the state of current research. Influence factors for the membership of different classes are individual (gender, occupational aspiration), environmental (academic field) and cognitive (university entrance score) variables. In contrast, family background is not found to be a strong influencing factor. While the positive effect of the cognitive factor as well as the occupational aspiration can be expected from former research, the effects of gender and academic field can be seen as additions of the current state of research that provide hypotheses for future research.

The two trajectories must be seen against the theoretical background of the “Four‐phase model of interest development” (Renninger & Hidi, 2019). The class "continuously high interest" seems to be able to maintain its continuously high interest. This group is permanently in the highest phases of the “Four‐phase model of interest development”. In contrast, the "descending interest" class already seems to be in a lower phase at the beginning of the study. In the further course of studies, a descent into a lower phase of the “Four‐phase model of interest development” seems to be characteristic for this class. It may only be possible to catch this group over a period of time with situational interest. Towards the end of their studies, when they are working towards their final thesis, many people move up again to a higher level.

The reasons for the downward trend of subject-interest is complex, but potential reasons for explanations can be offered. It is possible that the subject-interests shift to a practical application of the subject content, so that the reduced shift in interest results from this. Another explanation for this development of the class “descending interest” can be seen in the POI. The start of a study program is linked to a variety of changes for freshmen’s social and achievement situation. For example, requirements arise in regulating one’s learning process and achievement motivation as less individual support by universities is a characteristic of the new learning environment (Pillay & Ngcobo, 2010). Consequently, students have to cope with other tasks, which are often put before subject interests. The upward-trend before finishing the study program is possibly a result of acquiring grit.

Analyses of determinants for the membership in classes to answer research question 2 are in line with the current state of research. The strong effect of occupational aspiration to the class membership underline links between interest and academic choices. This replicates findings by Bargel et al. (1989) and Schiefele (2009). As already mentioned in the previous section, this analysis detects an effect of cognitive factors on interest. Results show that university entrance score is an important factor influencing class membership. However, people with a higher university entrance score are more likely to belong to the class “descending interest”. It is difficult to explain this result, but maybe there is a mismatch with the choice of the academic major or potentially too low demands in the study program. Against the background of the discussions in educational policy on interest in the STEM field and gender effects, the present results are likely to attract particular attention, especially in Germany (Gottfried et al., 2001; Krapp, 2018; Su et al., 2009). Findings in this study show that men as well as students from the academic fields engineering and social work more often belong to the class “continuously high interest” than women and students from other academic fields do.

Against the backdrop of the developmental trajectories of subject-interest and the influencing factors that affect it, new research questions occur that need to be addressed in the future. The question of the correlation between the development of subject-interests and research-based learning arises (Wessels et al., 2020). Furthermore, the influence the COVID-19 pandemic has on this research field as well as on the entire teaching at universities should be investigated (Ortiz, 2020). It remains open how far the research results can be generalised. Can the results, for example, also be transferred to traditional students or regions? Replication studies need to be initiated here. One possible answer could be that the result of this study can be generalized to other geographies with the following characteristics. Reinhard et al. (2016) emphasize a generalization of such results from cooperative education program especially to countries having a rich history in cooperative education programs, like South Africa or Namibia. Graf et al. (2014) propose a transfer of such results from cooperative education programs to other countries and education systems depending on the general economic conditions in a target country and a general interest in the expansion of the tertiary education sector, for instance through (education) policy reforms and initiatives in the target country. Against the background of research in learning and instruction, it would also be interesting to explore the correlation between learning difficulties and interest.

The present study has several limitations. Firstly, only students from a single university with twelve locations in one federal state of Germany were interviewed. Furthermore, this study is only able to use three academic majors in the datasets. The data is based on cooperative students that are recruited by the partner companies of the universities (Kupfer, 2013). Therefore, a generalization of the results is difficult so that it has to be replicated, for example with traditional students. This suggestion is already addressed in the last paragraph. Furthermore, the imprecise measurement of social origin could contribute to the fact that no significant effect was found here. Also, an influence of the different teaching methods in the three academic majors on interest cannot be ruled out and should be more investigated in further studies. Moreover, a large sample size is used here. In such situation it must be consider, that in large enough sample sizes even tiny and practically irrelevant effects become statistically significant.

However, the study also possesses several strengths. I am able to use panel data analyzing intraindividual changes of participants. Furthermore, I do research in higher education, which is very rare in the research field of interest. I am able to access extensive data from the university administration and to integrate it into analyses, which increases the quality of this study. The large sample size of 4,345 participants as well as the reliable measurements in the field study (ω ≥ 0.81) should also be positively emphasized. The use of innovative and complex analytical methods further underlines the importance of this study.

At this point, I would like to present practical implications that enable students to increase interest in their field of study. Even before they start their studies, students have the opportunity to match their interests with the subject they are studying. Online self-assessments are a successful method for this (Ćukušić et al., 2014). A further strengthening of subject-specific interests can be attained by focusing on the didactic offers of university lecturers. Based on targeted offers, the teaching quality can be increased, which can in turn push situational as well as individual interests (Biggs & Tang, 2011). A further practical implication to increase student’s interest and motivation or prevent burnout could be accompanying coaching programs at the beginning of their student program at universities. First approaches to implement this are available, but a more precise adaptation to the topic as well as to the target group is necessary (Unterbrink et al., 2012).

The results of this study build in important ways on the extant literature on the development of students’ subject-interest across the higher education years and offer important new findings. The present study can be used as a starting point for further research. I hope that the findings have produced the first important results for addressing the raised research question.

## Data Availability

Data is available. Please contact the corresponding author.

## Code Availability

Syntax is available. Please contact the corresponding author.

## References

[CR1] Ames C, Archer J (1988). Achievement goals in the classroom: Students' learning strategies and motivation processes. Journal of Educational Psychology.

[CR2] AusbildungPlus (2020). Duales Studium in Zahlen 2019. Trends und Analysen [Cooperative Education in Figures 2019. Trends and Analysis].

[CR3] Bargel T, Framheim-Peisert G, Sandberger JU (1989). Studienerfahrungen und studentische Orientierungen in den 80er Jahren. Trends und Stabilitäten [Study experiences and student orientations in the 1980s. Trends and Stabilities].

[CR4] Best H, Wolf C (2012). Comparing nested models and interpreting results from logit and probit regression. KZfSS Kölner Zeitschrift Für Soziologie Und Sozialpsychologie.

[CR5] Biggs J, Tang C (2011). Teaching for quality learning at University.

[CR6] Blossfeld H.─P, van Maurice J, Schneider T, Blossfeld H.─P, Roßbach H.─G (2019). The national educational panel study: need, main features, and research potential. Education as a lifelong process.

[CR7] Bong M (2001). Role of self-efficacy and task-value in predicting college students’ course performance and future enrolment intentions. Contemporary Educational Psychology.

[CR8] Chen FF (2007). Sensitivity of goodness of fit indexes to lack of measurement invariance. Structural Equation Modeling.

[CR9] Coertjens L, Donche V, De Maeyer S, van Daal T, Van Petgen P (2017). The growth trend in learning strategies during the transition from secondary to higher education in Flanders. Higher Educucation.

[CR10] Ćukušić M, Garača Z, Jadrić M (2014). Online self-assessment and students' success in higher education institutions. Computers & Education.

[CR11] Daniels Z (2008). Entwicklung schulischer Interessen im Jugendalter [Development of school interests in adolescence].

[CR12] Destatis (= Federal Statistical Office) (2020). Bildung und Kultur. Studierende an Hochschulen [Education and Culture. Students at universities].

[CR13] Deuer E, Meyer T, Walkmann R, Rahn S, Deuer E, Meyer T (2020). Das Studienverlaufspanel an der DHBW (2015–2019) [The study panel at the DHBW (2015–2019)]. Studienverlauf und Studienerfolg im Kontext des dualen Studiums [Study process and study success in cooperative education].

[CR14] Dotterer AM, McHale SM, Crouter AC (2009). The development and correlates of academic interests from childhood through adolescence. Journal of Educational Psychology.

[CR15] Durik AM, Vida M, Eccles JS (2006). Task values and ability beliefs as predictors of high school literacy choices: A developmental analysis. Journal of Educational Psychology.

[CR16] Eccles JS, Spence JT (1983). Expectancies, values, and academic behaviors. Achievement and achievement motives.

[CR17] Eccles JS, Midgley C, Wigfield A, Buchanan CM, Reuman D, Flanagan C, Mac Iver D (1993). Development during adolescence: The impact of stage-environment fit on young adolescents' experiences in schools and in families. American Psychologist.

[CR18] Fellenberg F, Hannover B (2006). Kaum begonnen, schon zerronnen? Psychologische Ursachenfaktoren für die Neigung von Studienanfängern, das Studium abzubrechen oder das Fach zu wechseln [Easy come, easy go? Psychological causes of students´ drop out of university or changing the subject at the beginning of their study]. Empirische Pädagogik.

[CR19] Fitzmaurice G, Davidian M, Verbeke G, Molenberghs G (2009). Longitudinal data analysis. Handbooks of modern statistical methods.

[CR20] Frenzel AC, Goetz T, Pekrun R, Watt HMG (2010). Development of mathematics interest in adolescence: Influences of gender, family, and school context. Journal of Research on Adolescence.

[CR21] Frenzel AC, Pekrun R, Dicke AL, Goetz T (2012). Beyond quantitative decline: Conceptual shifts in adolescents' development of interest in mathematics. Developmental Psychology.

[CR22] Gaspard H, Lauermann F, Rose N, Wigfield A, Eccles JS (2020). Cross-domain trajectories of students' ability self-concepts and intrinsic values in math and language arts. Child Development.

[CR23] Glover S, Dixon P (2004). Likelihood ratios: A simple and flexible statistic for empirical psychologists. Psychonomic Bulletin & Review.

[CR24] Gottfried AE, Fleming JS, Gottfried AW (2001). Continuity of academic intrinsic motivation from childhood through late adolescence: A longitudinal study. Journal of Educational Psychology.

[CR25] Graf L, Powell JJW, Fortwengel J, Bernhard N (2014). Dual study programmes in global context: Internationalisation in Germany and transfer to Brazil, France, Qatar, Mexico and the US.

[CR26] Grossnickle EM (2016). Disentangling curiosity: Dimensionality, definitions, and distinctions from interest in educational contexts. Educational Psychology Review.

[CR27] Guo J, Wang M-T, Ketonen EE, Eccles JS, Salmela-Aro K (2018). Joint trajectories of task value in multiple subject domains: From both variable- and pattern-centered perspectives. Contemporary Educational Psychology.

[CR28] Harackiewicz JM, Barron KE, Tauer JM, Elliot AJ (2002). Predicting success in college: A longitudinal study of achievement goals and ability measures as predictors of interest and performance from freshman year through graduation. Journal of Educational Psychology.

[CR29] Harackiewicz JM, Durik AM, Barron KE, Linnenbrink-Garcia L, Tauer JM (2008). The role of achievement goals in the development of interest: Reciprocal relations between achievement goals, interest, and performance. Journal of Educational Psychology.

[CR30] Hartinger A, Fölling-Albers M (2002). Schüler motivieren und interessieren – Ergebnisse aus der Forschung, Anregungen für die Praxis [Making pupils motivated and interested - Results from Research, Suggestions for Practice].

[CR31] Hauser RM, Tsai S-L, Sewell W-H (1983). A model of stratification with response error in social and psychological variables. Sociology of Education.

[CR32] Hidi S (2006). Interest: A unique motivational variable. Educational Research Review.

[CR33] Hidi S, Ainley M, Schunk DH, Zimmerman BJ (2008). Interest and self-regulation: Relationships between two variables that influence learning. Motivation and self-regulated learning: Theory, research, and application.

[CR34] Hidi S, Harackiewicz JM (2000). Motivating the academically unmotivated: A critical issue for the 21st century. Review of Educational Research.

[CR35] Hidi S, Renninger KA (2006). The four-phase model of interest development. Educational Psychologist.

[CR36] Hoffmann L (2002). Promoting girls' interest and achievement in physics classes for beginners. Learning and Instruction.

[CR37] Hoffmann L, Lehrke M (1986). Eine Untersuchung über Schülerinteressen an Physik und Technik [An investigation of students‘ interest in physics and technics]. Zeitschrift Für Pädagogik.

[CR38] Hoffmann L, Lehrke M, Todt E, Lehrke M, Hoffmann L, Gardner PL (1985). Development and change of pupils’ interest in physics: Design of longitudinal study (grade 5–10). Interests in science and technology.

[CR39] Høgheim S, Reber R (2019). Interesting, but less interested: Gender differences and similarities in mathematics interest. Scandinavian Journal of Educational Research.

[CR40] Honaker J, King G, Blackwell M (2011). Amelia II: A program for missing data. Journal of Statistical Software.

[CR41] Jung T, Wickrama KAS (2008). An introduction to latent class growth analysis and growth mixture modeling. Social and Personality Psychology Compass.

[CR42] Kim S, Klager C, Schneider B (2019). The effects of alignment of educational expectations and occupational aspirations on labor market outcomes: Evidence from NLSY79. The Journal of Higher Education.

[CR43] Krapp A, Krapp A, Prenzel M (1992). Das Interessenkonstrukt: Bestimmungsmerkmale der Interessenhandlung und des individuellen Interesses aus der Sicht einer Person-Gegenstands-Konzeption [The construct of interest: determinants of interest action and individual interest from the perspective of a person-object conception]. Interesse, Lernen, Leistung: Neuere Ansätze der pädagogisch-psychologischen Interessenforschung [Interest, Learning, Performance: Recent Approaches of Interest Research in Educational Psychological].

[CR44] Krapp A (2002). Structural and dynamic aspects of interest development: Theoretical considerations from an ontogenetic perspective. Learning and Instruction.

[CR45] Krapp A (2005). Basic needs and the development of interest and intrinsic motivational orientations. Learning and Instruction.

[CR46] Krapp A (2007). An educational–psychological conceptualisation of interest. International Journal for Educational and Vocational Guidance.

[CR47] Krapp A, Rost DH, Sparfeldt JR, Buch SR (2018). Interesse [Interest]. Handwörterbuch Pädagogische Psychologie [Handbook of Educational Psychology].

[CR48] Kupfer F (2013). Duale Studiengänge aus Sicht der Betriebe: Praxisnahes Erfolgsmodell durch Bestenauslese [Cooperative education programmes from the point of view of the companies: Practical success model through selection of the best]. Berufsbildung in Wissenschaft und Praxis – BWP.

[CR49] Kyndt E, Coertjens L, van Daal T, Donche V, Gijbels D, Van Petegem P (2015). The development of students' motivation in the transition from secondary to higher education: A longitudinal study. Learning and Individual Differences.

[CR50] Kyndt E, Donche V, Coertjens L, van Daal T, Gijbels D, Van Petegem P (2019). Does self-efficacy contribute to the development of students’ motivation across the transition from secondary to higher education?. European Journal of Psychology of Education.

[CR51] Liebendörfer M, Schukajlow S (2017). Interest development during the first year at university: Do mathematical beliefs predict interest in mathematics?. ZDM.

[CR52] Little RJ (1988). A test of missing completely at random for multivariate data with missing values. Journal of the American Statistical Association.

[CR53] Loewenstein G (1994). The psychology of curiosity: A review and reinterpretation. Psychological Bulletin.

[CR54] Maurice J, Dörfler T, Artelt C (2014). The relation between interests and grades: Path analyses in primary school age. International Journal of Educational Research.

[CR55] McDonald RP (1999). Test theory: A unified treatment.

[CR56] Mood C (2010). Logistic regression: Why we cannot do what we think we can do, and what we can do about it. European Sociological Review.

[CR57] Musu-Gillette LE, Wigfield A, Harring JR, Eccles JS (2015). Trajectories of change in students’ self-concepts of ability and values in math and college major choice. Educational Research and Evaluation.

[CR58] Neugebauer M, Heublein U, Annabel D (2019). Higher education dropout in Germany: Extent, causes, consequences, prevention. Zeitschrift Für Erziehungswissenschaft.

[CR59] Ortiz PA (2020). Teaching in the time of COVID-19. Biochemistry and Molecular Biology Education: A Bimonthly Publication of the International Union of Biochemistry and Molecular Biology.

[CR60] Pany P, Lörnitzo A, Auleitner L, Heidinger C, Lampert P, Kiehn M (2019). Using students’ interest in useful plants to encourage plant vision in the classroom. Plants, People, Planet, Special Issue: Standing in the Shadows of Plants: New Perspectives on Plant Blindness.

[CR61] Pillay AL, Ngcobo HSB (2010). Sources of stress and support among rural-based first-year university students: An exploratory study. South African Journal of Psychology.

[CR62] Protsch P, Solga H (2015). How employers use signals of cognitive and noncognitive skills at labour market entry: Insights from field experiments. European Sociological Review.

[CR63] Putnick DL, Bornstein MH (2016). Measurement Invariance conventions and reporting: The state of the art and future directions for psychological research. Developmental Review: DR.

[CR64] Ram N, Grimm KJ (2009). Methods and measures: Growth mixture modeling: A method for identifying differences in longitudinal change among unobserved groups. International Journal of Behavioral Development.

[CR65] Reinecke J (2006). Longitudinal analysis of adolescents’ deviant and delinquent behavior. Methodology.

[CR66] Reinhard K, Pogrzeba A, Townsend R, Pop AP (2016). A comparative study of cooperative education and work integrated learning in Germany, South Africa, and Namibia. Asia-Pacific Journal of Cooperative Education.

[CR67] Renninger KA, Hidi S (2016). The power of interest for motivation and engagement.

[CR68] Renninger K, Hidi S, Renninger K, Hidi S (2019). Interest development and learning. The Cambridge handbook of motivation and learning.

[CR69] Renninger KA, Ewen L, Lasher AK (2002). Individual interest as context in expository text and mathematical word problems. Learning and Instruction.

[CR70] Richard FD, Bond CF, Stokes-Zoota JJ (2003). One hundred years of social psychology quantitatively described. Review of General Psychology.

[CR71] Richardson M, Abraham C, Bond R (2012). Psychological correlates of university students' academic performance: A systematic review and meta-analysis. Psychological Bulletin.

[CR72] Rotgans JI, Schmidt HG (2011). Situational interest and academic achievement in the active-learning classroom. Learning and Instruction.

[CR73] Sabates, R., Harris, A. L., & Staff, J. (2011). Ambition gone awry: The long-term socioeconomic consequences of misaligned and uncertain ambitions in adolescence. *Social Science Quarterly, 92*(4)*,* 959–977. Retrieved June 18, 2021, from http://www.jstor.org/stable/42956558 22180878

[CR74] Sansone C, Thoman DB (2005). Interest as the missing motivator in self-regulation. European Psychologist.

[CR75] Scherrer V, Preckel F, Schmidt I, Elliot AJ (2020). Development of achievement goals and their relation to academic interest and achievement in adolescence: A review of the literature and two longitudinal studies. Developmental Psychology.

[CR76] Schiefele U, Wentzel KR, Wigfield A (2009). Situational and individual interest. Handbook of motivation at school.

[CR77] Schiefele U, Krapp A, Schreyer I (1993). Metaanalyse des Zusammenhangs von Interesse und schulischer Leistung [Meta-analysis of interest and academic achievement]. Zeitschrift Für Entwicklungspsychologie Und Pädagogische Psychologie.

[CR78] Schiefer IM, Becker S, Artelt C (2018). A person centered approach for analyzing the development of students’ subject-interests in language arts, Mathematics, and English as a foreign language from Grade 4 to 11. Psychologie in Erziehung Und Unterricht.

[CR79] Schnettler T, Bobe J, Scheunemann A, Fries S, Grunschel C (2020). Is it still worth it? Applying expectancy-value theory to investigate the intraindividual motivational process of forming intentions to drop out from university. Motivation and Emotion.

[CR80] Sewell W-H, Haller AO, Portes A (1969). The educational and early occupational attainment process. American Sociological Review.

[CR81] Su R, Rounds J, Armstrong PI (2009). Men and things, women and people: A meta-analysis of sex differences in interests. Psychological Bulletin.

[CR82] Tabachnick BG, Fidell LS (2013). Using multivariate statistics.

[CR83] Thiel, F., Veit, S., Blüthmann, I., Lepa, S. & Ficzko, M. (2008). Ergebnisse der Befragung der Studierenden in den Bachelorstudiengängen an der Freien Universität Berlin - Sommersemester 2008 [Results of students in Bachelor's degree programmes survey at Freie Universität Berlin - Summer term 2008]. Retrieved February 27, 2021, from https://www.geo.fu-berlin.de/studium/Qualitaetssicherung/Ressourcen/FU_bachelorbefragung_2008.pdf

[CR84] Todt E, Arbinger R, Seitz H, Wildgrube W (1974). Untersuchungen über die Motivation zur Beschäftigung mit naturwissenschaftlichen Problemen (Sekundarstufe I: Klassen 5–9) [Studies on the motivation to work with science problems (secondary level: Grades 5–9)].

[CR85] Unterbrink T, Pfeifer R, Krippeit L, Zimmermann L, Rose U, Joos A, Hartmann A, Wirsching M, Bauer J (2012). Burnout and effort-reward imbalance improvement for teachers by a manual-based group program. International Archives of Occupational and Environmental Health.

[CR86] Vogler-Ludwig K, Düll N, Kriechel B (2016). Arbeitsmarkt 2030. Wirtschaft und Arbeitsmarkt im digitalen Zeitalter. Prognose 2016. Kurzfassung. [Labour market 2030. Economy and labor market in the digital age. Prognosis 2016. Summary].

[CR87] Wang MT, Chow A, Degol JL, Eccles JS (2017). Does everyone's motivational beliefs about physical science decline in secondary school?: Heterogeneity of adolescents' achievement motivation trajectories in physics and chemistry. Journal of Youth and Adolescence.

[CR88] Wessels I, Rueß J, Gess C, Deicke W, Ziegler M (2020). Is research-based learning effective? Evidence from a pre–post analysis in the social sciences. Studies in Higher Education.

[CR89] Wild S, Alvarez S (2020). Cooperative education in the higher education system and Big Five personality traits in Germany. International Journal of Work-Integrated Learning.

[CR90] Wild S, Neef C (2019). The role of academic major and academic year for self-determined motivation in cooperative education. Industry and Higher Education.

[CR91] Wild, S., & Schulze Heuling, L. (2020). Student dropout and retention: an event history analysis among students in cooperative higher education. *International Journal of Educational Research, 104*. 10.1016/j.ijer.2020.101687

[CR92] Xu C, Lern S, Onghena P (2021). Examining developmental relationships between utility value, interest, and cognitive competence for college statistics students with differential self-perceived mathematics ability. Learning and Individual Differences.

[CR93] Yates S, Harris A, Sabates R, Staff J (2011). Early Occupational Aspirations andFractured Transitions: A Study of Entry into‘NEET’ Status in the UK. Journal of Social Policy.

